# Rhythmic and Periodic Patterns and Seizures in Hypoxic–Ischemic Encephalopathy

**DOI:** 10.1097/WNP.0000000000001235

**Published:** 2025-12-24

**Authors:** Pia De Stefano, Nicolas Gaspard

**Affiliations:** * Neuro-Intensive Care Unit, Department of Intensive Care, University Hospital of Geneva, Geneva, Switzerland;; † EEG & Epilepsy Unit, Department of Clinical Neurosciences, University Hospital of Geneva, Geneva, Switzerland;; ‡ Department of Neurology, Hôpital Universitaire de Bruxelles-Hôpital Erasme, Université Libre de Bruxelles, Brussels, Belgium;; § Laboratory of Experimental Neurology, Université Libre de Bruxelles, Brussels, Belgium; and; ‖ Department of Neurology, Yale University School of Medicine, New Haven, CT

**Keywords:** cardiac arrest, hypoxic ischemic encephalopathy, postanoxic status epilepticus, Rhythmic, periodic patterns RPPs

## Abstract

Cardiac arrest is a major cause of hypoxic–ischemic brain injury, often resulting in coma even after the return of spontaneous circulation. In this severely ill population, accurate neurologic prognostication is essential for guiding treatment decisions. Continuous electroencephalography (EEG) has become a cornerstone of postcardiac arrest care, offering real-time monitoring of cerebral activity and facilitating early detection of electrographic seizures and other pathologic patterns. This narrative review focuses on the standardized interpretation of EEG findings using the 2021 American Clinical Neurophysiology Society Critical Care EEG terminology. Key EEG patterns—such as generalized periodic discharges, suppression–burst, and status epilepticus (SE)—are discussed in the context of their prognostic significance and therapeutic implications. Particular attention is given to the progressive restoration of background EEG continuity and amplitude over the first 24 hours after the arrest, which is increasingly recognized as a marker of the extent of neuronal damage and potential for recovery. Seizure and SE management remains complex in comatose postcardiac arrest patients, with recent studies highlighting variable outcomes and the potential risks of both under- and overtreatment. We underscore the importance of individualized treatment strategies informed by EEG characteristics and integrated with clinical examination, neuroimaging, and serum biomarkers. Multimodal prognostication helps identify patients with a potential for meaningful recovery while avoiding unnecessary interventions in those with poor neurologic prognosis. EEG-guided care is crucial in optimizing outcomes after cardiac arrest and enhancing the precision of neurocritical care.

Cardiac arrest is the major cause of hypoxic–ischemic brain injury,^1^ which significantly contributes to mortality and severe neurologic disability worldwide.^2^ Despite advances in cardiopulmonary resuscitation and postresuscitation care, many patients who achieve return of spontaneous circulation (ROSC) remain comatose and require support of vital functions and neurologic prognostication. The American Heart Association emphasizes the importance of postcardiac arrest care, which includes hemodynamic support, mechanical ventilation, temperature management, and the diagnosis and treatment of seizures to mitigate brain injury.^3,4^ The American Academy of Neurology also highlights the critical role of neuroprotective interventions and the need for accurate prognostication to improve outcomes.^5^

Prognostication after cardiac arrest involves a multimodal approach, including clinical neurologic examinations, brain imaging, electrophysiologic tests, and serum biomarkers. Key indicators of severe hypoxic–ischemic brain injury include the absence of corneal and pupillary reflexes, unfavorable EEG patterns, high levels of neuron-specific enolase, and signs of diffuse brain injury on imaging.^6,7^ Current guidelines from the American Heart Association^4^ and European Resuscitation Council and European Society of Intensive Care (ERC/ESICM) 2021^7^ and 2025^8^ recommend delaying prognostication until at least 72 hours after ROSC to allow for the clearance of sedative drugs and to minimize the risk of falsely pessimistic predictions. With this precaution, the presence of at least two indicators of severe hypoxic–ischemic brain injury is associated with no chance of meaningful recovery.

In this context, continuous EEG monitoring has become a cornerstone of postcardiac arrest management, providing real-time assessment of brain function, early detection of seizures, and important prognostic information. However, the interpretation of EEG in this critically ill population is often challenging, requiring familiarity with standardized terminology, evolving patterns, and their clinical implications.

The American Clinical Neurophysiology Society (ACNS) has developed and updated a standardized terminology for EEG interpretation in critical care settings, with the latest version published in 2021.^9^ This nomenclature allows the consistent description of EEG patterns such as generalized periodic discharges (GPDs), suppression–burst patterns, and seizures, which frequently occur in the postcardiac arrest setting. Recognizing and appropriately classifying these patterns is essential, as they can provide insight into the severity of brain injury and guide management decisions. Particular attention is needed to distinguish encephalopathic patterns from ictal phenomena, as well as to understand the nuances of patterns that lie along the ictal–interictal continuum (IIC).^9^ Beyond static pattern recognition, the temporal evolution of EEG findings plays a critical role in prognostication. Serial EEG assessments, rather than single time point evaluations, enhance the accuracy of prognostication, especially when integrated with clinical examination findings, neuroimaging, and biomarkers.

Seizures and status epilepticus (SE) are detected in up to 27%^10,11^ comatose survivors undergoing continuous EEG monitoring.^12–14^ The management of seizures and SE postcardiac arrest remains controversial. Although traditional approaches advocate for aggressive antiseizure treatment,^15^ other studies, including the randomized controlled Treatment of ELectroencephalographic STatus epilepticus After cardiopulmonary Resuscitation Trial (TELSTAR), have questioned whether such interventions improve outcomes in all patients.^16^ This raises important clinical dilemmas: although seizures and SE occurring in the setting of a continuous EEG background may be treatable and compatible with recovery, those arising from suppressed background or suppression–burst mostly indicate extensive brain injury and carry a grim prognosis.^17–20^

These findings highlight the need for individualized, context-sensitive treatment strategies, balancing the potential benefits of seizure control against the risks of overtreatment in patients with severe anoxic injury, as revealed from a recent study from our group.^21^

In this narrative review, we aim to summarize the interpretation of EEG findings in comatose survivors of cardiac arrest, emphasizing standardized terminology the significance of background EEG features, the temporal evolution of patterns, and the management of seizures and SE. By integrating current evidence and guidelines, we aim to provide actionable clinical strategies for neurologists, intensivists, and epileptologists involved in the care of this vulnerable patient population.

## EEG NOMENCLATURE IN POSTANOXIC PATIENTS

Postanoxic brain injury is characterized by unique and often complex EEG patterns, whose interpretation is helped by standardized interpretation. According to the 2021 ACNS terminology, EEG patterns in critically ill patients are categorized based on three principal attributes: pattern morphology, location, and frequency.

### Rhythmic and Periodic Patterns

Periodic patterns are defined by the repetition of waveforms at nearly regular intervals with an intervening period of relative quiescence, whereas rhythmic patterns consist of continuous repetition of uniform waveforms without an interrupting visible background.

**FIG. 1.**
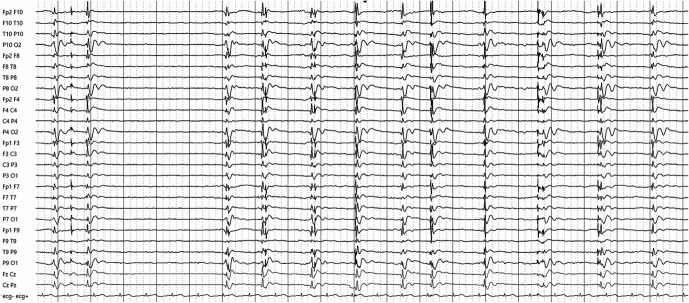
Epoch of 20 s of EEG displayed in a bipolar longitudinal montage. 1-Hz GPD on a suppressed background. Pattern typically associated with poor outcome. Filter setting: high-pass 1 Hz, low-pass 70 Hz.

A key periodic pattern of interest following cardiac arrest are generalized periodic discharges (GPDs), which are characterized by bilaterally synchronous and symmetric periodic discharges. By definition, discharges last <0.5 seconds, regardless of number of phases, or, if they last ≥0.5 seconds, have no more than 3 phases, as opposed to bursts, which are defined as lasting ≥0.5 seconds and having at least 4 phases.^9^ Continuous GPDs may present on a continuous or suppressed background (Fig. 1), with different prognostic and therapeutic significance.^22^

Although GPDs do not constitute electrographic seizures by themselves, those that have a frequency exceeding 2.5 Hz, show evolution in frequency, amplitude, or morphology, or are associated with clinical signs fulfil criteria for an ictal phenomenon.^9,23^ However, in the comatose postcardiac arrest patient, GPDs are typically observed at frequencies up to 2.5 Hz^16,24^ and often lie along the Ictal–Interictal Continuum (IIC).

### Ictal–Interictal Continuum

The Ictal–Interictal Continuum is a clinically significant but challenging situation. Patterns along the IIC possess features that are intermediate between clearly interictal abnormalities and definite seizures. Examples include GPDs at 1 to 2.5 Hz without evolution, or GPDs at more than 0.5 Hz with a “plus” modifier (superimposed rhythmic delta or fast activity) or fluctuation in frequency, morphology, or spatial distribution. These patterns are not invariably ictal but carry a heightened risk of seizures and may be associated with ongoing brain injury. In clinical practice, identifying whether a pattern on the IIC represents a seizure often requires evaluation of additional factors, including temporal evolution, the presence of a clinical correlate (such as subtle myoclonus or autonomic changes), or responsiveness to an empirical trial of antiseizure medication (ASM).^25^ A transient improvement in the EEG pattern following ASM administration is not sufficient to confirm an ictal nature.^25^ Unequivocal demonstration of the ictal nature requires both EEG and clinical improvement (increase in the consciousness level), but this is almost always impossible to judge in a population that is, by definition, comatose.

### Suppression–Burst

Another pattern commonly encountered is suppression–burst (also called burst–suppression). According to the ACNS 2021 terminology,^9^ is defined as periods of relatively high-voltage electrical activity (“bursts”) alternating with periods of very low amplitude of suppressed activity (“suppressions”), typically below 10 µV in amplitude, with periods of suppression constituting at least 50% of the recording. Bursts are defined as any EEG activity lasting at least 0.5 seconds, clearly distinguishable from the suppression or background, and typically of higher amplitude than the intervening suppressed periods.^9^ Differently, discharges refer to paroxysmal waveforms that are clearly distinguishable from the background, lasting less than 0.5 seconds, and may be single or repetitive. Discharges can be further characterized by their morphology (e.g., spikes, sharp waves, spike-and-wave complexes) and may occur in isolation or in a periodic or rhythmic pattern.^9^

**FIG. 2.**
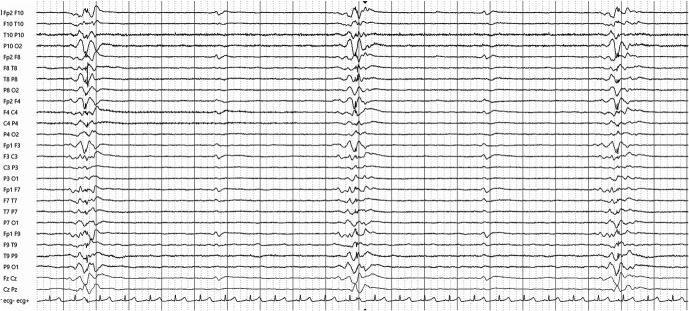
Epoch of 20 s of EEG displayed in a bipolar longitudinal montage. Burst–suppression with identical bursts. Pattern typically associated with poor outcome. Filter setting: high-pass 1 Hz, low-pass 70 Hz.

In patients recovering from cardiac arrest, spontaneous suppression–burst patterns have historically been associated with severe cortical injury and poor prognosis^26,27^ (Fig. 2**)**. However, this should be interpreted within the broader clinical context, taking into account the effects of sedative medications. Nevertheless, standard sedative doses do not meaningfully affect EEG prognostic markers.^28^ Recently, studies have drawn the attention to features that help discriminate spontaneous postanoxic bursts from drug-induced bursts, such as morphologic interburst similarity and interhemispheric synchrony and symmetry.^27,29–32^ Identical bursts are defined as bursts within a suppression–burst pattern with a highly stereotyped morphology, such that consecutive bursts are visually similar with one another throughout the recording. These bursts are typically bilateral and synchronous, and their similarity is quantifiable by a high cross-correlation coefficient (commonly >0.75) between bursts.^27,30^

### Seizures and Status Epilepticus

The identification of electrographic seizures and electrographic SE is essential in the comatose cardiac arrest population. Based on ACNS 2021 criteria,^9^ which include the Salzburg criteria for nonconvulsive SE,^23^ an electrographic seizure is defined by the presence of epileptiform discharges averaging more than 2.5 Hz for at least 10 seconds (i.e., more than 25 discharges over any 10-second epoch), or rhythmic/periodic activity with unequivocal evolution in frequency, morphology, or spatial distribution lasting at least 10 seconds. Electrographic SE is diagnosed when there is continuous electrographic seizure activity lasting at least 10 minutes, or intermittent seizures totaling at least 20% of each 1-hour period recording window. Despite the use of those criteria, it is worth mentioning that Salzburg criteria were derived and validated from a nonhypoxic epilepsy population.^23,33^

Importantly, electrographic seizures and SE after cardiac arrest may lack any clinical correlate, particularly in deeply comatose, sedated, or paralyzed patients (despite the use on neuromuscular agents in postcardiac arrest patients is rare), and they are reported in 1% to 20% of postcardiac arrest patients,^34^ underscoring the essential role of continuous EEG monitoring. However, postcardiac arrest patients may also present with electroclinical SE, defined as a clinical correlate that is time-locked with any EEG pattern.^9^ One of the most frequent electroclinical SE in this setting is myoclonic SE, defined as a myoclonus time-locked with any EEG pattern, which should be differentiated from status myoclonus without EEG correlates. Another important distinction to make is between myoclonic SE on a (nearly) continuous background and myoclonic SE on a suppressed background, as the outcome and the therapeutic significances are different.^35^

In light of these findings, status myoclonus, defined only clinically without EEG correlate and associated background, is no longer listed among the poor outcome predictors in the new 2025 American Heart Association Guidelines on Post-Cardiac Arrest Care.^4^

### Temporal Evolution of EEG Patterns and the Role of Background Activity

The temporal evolution of EEG patterns following cardiac arrest provide critical information regarding the severity of anoxic brain injury and inform neurologic prognostication. Continuous EEG monitoring during the first 24 h after arrest captures dynamic changes that can either reflect recovery or progressive brain injury. Careful analysis of the background EEG activity, including its continuity, amplitude, and frequency, is essential for accurate interpretation and outcome prediction.^36,37^

Background continuity is, in fact, a fundamental prognostic marker.

Immediately following cardiac arrest, the EEG often exhibits a profoundly suppressed background or an isoelectric pattern, reflecting complete, but potentially reversible, synaptic failure^28^; however, in some patients, neurons may already be depolarized. Over time, recovery of the EEG background toward continuous normal amplitude activity within 12 to 24 h after ROSC is associated with favorable outcomes,^10,28,38^ whereas persistence of suppression beyond the first 24 hours or evolution toward spontaneous bilateral synchronous bursts or a burst–suppression with identical bursts or an otherwise suppressed EEG portends a poor prognosis.^19,28,39,40^

In a study by Cloostermans et al., patients with a continuous EEG background at 24 hours had significantly better outcomes compared with those with persistent low-voltage or isoelectric patterns. More recent data from large cohort studies support these findings, reinforcing that early restoration of continuity remains among the most reliable predictors of favorable prognosis.^10,36,37^

The presence of epileptiform activity, such as GPDs, seizures, or SE, carries a more nuanced prognostic implication.

Early (<24 h) appearance of periodic discharges on a suppressed background or their emergence from a spontaneous suppression–burst pattern is associated with worse outcome than their later appearance after restoration of continuous normal amplitude background EEG activity.^21,36^ These divergent trajectories might reflect different scenarios of synaptic recovery, with incomplete recovery leading to excitation–inhibition imbalance and cortical hyperexcitability before normally modulated activity can emerge.^41^

**FIG. 3.**
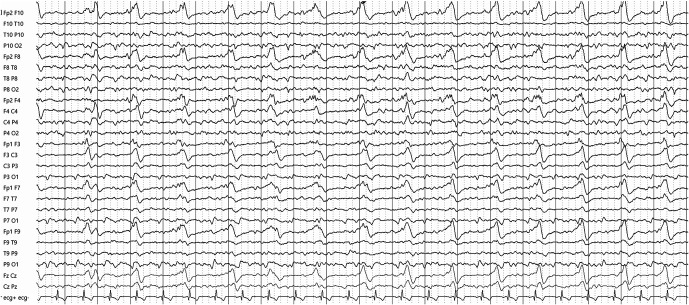
Epoch of 20 s of EEG displayed in a bipolar longitudinal montage. 1-Hz GPD on a continuous background. Pattern associated with an indeterminate outcome. Filter setting: high-pass 1 Hz, low-pass 70 Hz.

**FIG. 4.**
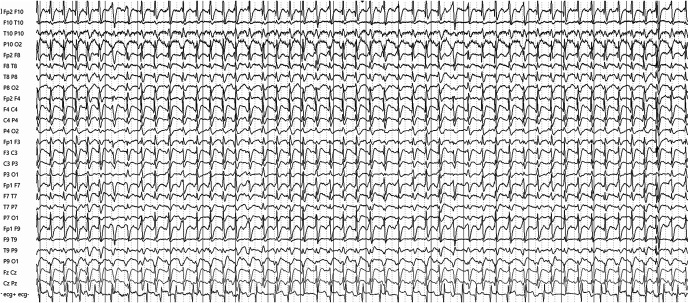
Epoch of 20 s of EEG displayed in a bipolar longitudinal montage. Definite electrographic status epilepticus without a visible background between discharges. Pattern typically associated with poor outcome. Filter setting: high-pass 1 Hz, low-pass 70 Hz.

**FIG. 5.**
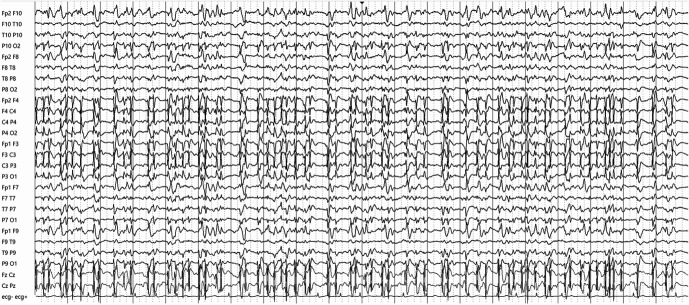
Epoch of 20 s of EEG displayed in a bipolar longitudinal montage. Definite electrographic status epilepticus with visible background between discharges. Pattern associated with an indeterminate outcome. Filter setting: high-pass 1 Hz, low-pass 70 Hz.

**FIG. 6.**
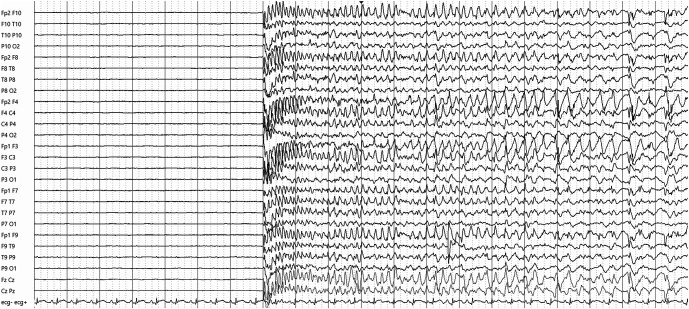
Epoch of 20 s of EEG displayed in a bipolar longitudinal montage. Suppression-seizure: seizure emerging from a suppressed background. Pattern typically associated with poor outcome. Filter setting: high-pass 1 Hz, low-pass 70 Hz.

Similarly, seizures occurring in a background of continuous EEG may not invariably predict poor outcome and may even be compatible with neurologic recovery.^10,15,20,21^ In contrast, seizures arising from a highly suppressed or burst-suppressed background generally reflect severe cortical injury and are associated with unfavorable prognosis, despite aggressive treatment^21,26–28,40^ (Figs. 3–6).

EEG reactivity to auditory, tactile, or noxious stimuli, defined as a clear and reproducible change in amplitude or frequency in response to stimulation, is widely regarded as a portent of good prognosis.^37,42^ However, if the EEG background is already (nearly) continuous within 12 to 24 hours after cardiac arrest, an EEG pattern with a sensitivity of approximately 50% and specificity of 90 to 95% for good neurologic outcome, the presence of EEG reactivity does not provide additional prognostic value. This is supported by large prospective studies showing that early continuous EEG background itself is a strong predictor of good outcome, and that adding EEG reactivity assessment in this context does not significantly improve predictive accuracy.^28,43^ The American Heart Association also notes that although EEG reactivity is associated with outcome, its incremental value is limited when the background is already continuous and favorable^44^ and in the very recent 2025 American Heart Association Guidelines on Post-Cardiac Arrest Care, it is recommended not to use the absence of EEG reactivity within 72 h to support the prognosis of unfavorable neurologic outcome.^4^

In a substantial number of critically ill patients, stimulation will bring out stimulus-induced periodic, rhythmic, or ictal discharges a phenomenon sometimes referred to as “SIRPIDs.”^45^ SIRPIDs consist of rhythmic, periodic, or ictal-appearing discharges that are consistently elicited by external stimulation, such as tactile, auditory, or painful stimuli, and SIRPIDs are associated with other EEG abnormalities such as GPDs and may coexist with spontaneous electrographic seizures.^45^ Although not regarded as normal reactivity, SIRPIDs nonetheless represent a form of cortical response to external stimuli, but, in postanoxic coma, their clinical and prognostic significance is unclear.^46,47^

Another not infrequent and striking pattern in postanoxic coma is stimulus-induced bursts, in which bursts, which can also occur spontaneously, are consistently evoked by external stimulation. Although they unmistakably denote at least partial integrity of ascending arousal networks, they are nonetheless associated with a poor outcome.^48^

### Seizure and Status Epilepticus Management: Evidence for Antiseizure Treatments and Limitations of Current Approaches

Seizures and SE occur in up to 27%^10,11^ patients following cardiac arrest and are associated with significant morbidity and mortality.^12–14^ Management strategies for these events remain complex and should be guided by the estimated severity of the underlying brain injury, as assessed by the EEG background, and the presence or absence of recovery signs. As seizures and SE are commonly purely electrographic in comatose patients after cardiac arrest, their detection requires continuous EEG monitoring.^34^

The decision to initiate ASM must consider both the seizure burden and the background EEG pattern. Seizures and SE occurring in the context of a continuous, normal voltage EEG background have been associated with better potential for recovery, and may benefit from protracted.^10,21^ Conversely, seizures and SE arising from a suppressed or suppression–burst background are typically markers of severe and irreversible brain injury, and aggressive treatment will not improve outcome.^26–28,40^

Current guidelines suggest treating unequivocal seizures and SE after cardiac arrest with standard ASMs, although the quality of evidence remains low.^7^ Common first-line agents include benzodiazepines (e.g., midazolam, diazepam, clonazepam, or lorazepam), followed by loading doses of nonsedating ASM such as levetiracetam, valproic acid, or lacosamide.^49^ In refractory cases, anesthetic agents such as propofol, midazolam infusions, barbiturates, or ketamine may be used to achieve EEG or SE suppression, although this approach carries a risk of additional complications, including hypotension and immunosuppression.^3,49^

Emerging data challenge the assumption that aggressive suppression of all rhythmic and periodic patterns improves outcome. The TELSTAR randomized controlled trial investigated whether aggressive antiseizure treatment of rhythmic and periodic EEG patterns after cardiac arrest improved neurologic outcomes and found no significant difference between treated and untreated groups.^16^ Specifically, targeting suppression of GPDs and other nonevolving rhythmic patterns with deep sedation and multiple ASMs did not lead to improved rates of good neurologic recovery. However, for subgroups with patterns fulfilling ACNS criteria^9^ for electrographic SE, outcomes in the intervention group were better than in the control group.^16^ The trial was underpowered for these subgroup analyses; however, these results suggest that prolonged antiseizure treatment of patients with postanoxic SE may improve outcome.

Moreover, the trial neither enrolled nor stratified patients based on the severity of brain injury as assessed by ERC/ESICM 2021 multimodal prognostic tools,^7^ potentially obscuring treatment effects in patients with milder forms of postanoxic encephalopathy. These limitations led to conduct a pooled analysis including 274 individual patients from three databases, including TELSTAR, which confirmed that EEG patterns meeting criteria for definite SE or possible SE are not necessarily related to a poor outcome of comatose CA survivors: the probability of a good neurologic outcome at three months was 25% in patients with ≤2 indicators of severe injury according to ERC/ESICM guidelines. Patients with a good outcome also had a higher discharge frequency, a discontinuous or continuous background pattern (but not suppression–burst or suppression) before SE onset and received higher ASMs doses, whereas semiology (motor vs. nonconvulsive) did not influence prognosis.^21^ This is another important finding from this study that challenges the notion that myoclonic status epilepticus is invariably a poor outcome scenario.

Despite these interesting findings, the benefit of aggressive ASM treatment remains limited. The ongoing randomized prospective multicenter Treatment of Electrographic Status Epilepticus after Cardiopulmonary Resuscitation (TELSTAR)-II trial currently investigates whether electrographic SE treatment improves outcome of comatose patients after cardiac arrest (www.telstartrial.nl).

An important limitation in current management strategies is the difficulty in differentiating harmful seizures from “epiphenomena” of severe brain injury. Periodic patterns such as GPDs without evolution may represent an epiphenomenon rather than ictal activity, making the decision to treat more challenging. Moreover, antiseizure therapies carry risks, including sedation, hypotension, and extend in-hospital stay with prolonged mechanical ventilation, all factors that may themselves negatively impact recovery. Therefore, clinicians must carefully weigh the potential benefits of seizure control against the risks of overtreatment, particularly when prognostic indicators suggest a poor likelihood of neurologic recovery.

Several expert consensus recommendations advise focusing antiseizure therapy primarily on patients with favorable prognostic signs, such as preserved brainstem reflexes and a continuous EEG background during and before SE.^10,19,21,50^ As recovery of a background continuity in the first 24 h is a key factor in the neuroprognostication management and treatment decision, it is crucial to EEG monitor these patients early.^21,36^

In patients with a suppression–burst or suppressed background, especially if associated with absent brainstem reflexes and bilateral absence of SSEPs, seizure management may reasonably be limited to comfort measures. Future directions include efforts to better stratify patients based on EEG characteristics, biomarkers, and imaging to guide individualized antiseizure therapy. Trials exploring newer agents, the benefit of aggressive treatment (TELSTAR-II), neuroprotective strategies, and personalized EEG-based interventions are ongoing and may offer more targeted approaches to managing seizures after cardiac arrest.

## CONCLUSIONS

Overall, a structured approach to EEG interpretation using the ACNS 2021 Critical Care EEG terminology allows for more reproducible, clinically actionable classification of EEG findings after cardiac arrest. Clinicians should remain vigilant for the presence of evolving periodic or rhythmic patterns, spontaneous suppression–burst activity, and electrographic seizures and SE, all of which carry prognostic implications. Interpretation must always integrate the EEG findings with the patient's clinical context, timing relative to cardiac arrest, and the broader multimodal prognostic framework.

The temporal evolution of EEG patterns, particularly the restoration of background continuity in the first 24 h, plays a central role in neurologic prognostication after cardiac arrest.

Finally, the decision to initiate antiseizure therapy in the presence of electrographic seizures and SE in the postcardiac arrest setting must be individualized based on the EEG background, in particular before the emergence of the SE, clinical context, and multimodal prognostic indicators, acknowledging that the evidence for improved outcomes with antiseizure therapy in this setting is still limited.

A balanced, patient-centered approach is essential to optimize outcomes in those patients with definite SE who may benefit from protracted intensive care treatment, while minimizing futile overtreatment.
